# Knowledge and Experiences of Health Professionals in the Peripheral Management of Leishmaniasis in Morocco (ELHajeb)

**DOI:** 10.1155/2020/8819704

**Published:** 2020-09-15

**Authors:** K. El-Mouhdi, M. Fekhaoui, F. Elhamdaoui, H. Guessioui, A. Chahlaoui

**Affiliations:** ^1^Geo-Biodiversity and Natural Patrimony Laboratory, GEOPAC Center, Scientific Institute, Mohammed V University in Rabat, Morocco; ^2^Scientific Institute, Mohammed V University in Rabat, Morocco; ^3^Higher Institute of Nursing Professions and Health Techniques Meknes, Regional Health Directorate Fes-Meknes, Ministry of Health, Morocco; ^4^Natural Resources Management and Development Team, Laboratory of Health and Environment, Faculty of Sciences, Moulay Ismail University, Meknes, Morocco

## Abstract

**Background:**

Morocco hopes to eliminate leishmaniases by 2030. These diseases exist in cutaneous and visceral forms and constitute a serious public health problem. The fight against these parasitoses is carried out within the framework of a national program to control leishmaniases, which offers free treatment. However, the screening rate in public health structures does not exceed 35%.

**Objective:**

To determine the knowledge and experience of the social actors directly involved in the fight against leishmaniasis to contribute to analyse and understand the factors of this underreporting and to draw scientific recommendations to improve screening and control activities.

**Methods:**

Using a self-administered questionnaire, we conducted an exploratory survey during April and May 2019 among all health professionals working in public health structures in the province of ELHajeb in central Morocco.

**Results:**

We found that most of the health professionals had good knowledge about the clinical signs of each form of leishmaniasis, but they had erroneous information about the true vector of the disease, the reservoirs, and the mode of transmission. 76% recognized the national leishmaniases control program and only 17% received continuing education. 85% of these professionals focused on the curative aspects of the program. 47% stated that patients do not adhere to the antileishmaniasis treatment, and 25% stated that the population uses the concept of “Hboub of Chniwla” to refer to cutaneous leishmaniasis.

**Conclusion:**

The study concluded that the operationalization of the activities of the leishmaniases control program recognizes some weaknesses which explain the underscreening of cases. Improvement of this situation requires the implementation of continuous training programs for caregivers and awareness-raising programs for citizens which should focus on the mode of transmission, preventive measures against reservoirs, sand flies bites, and recognition of lesions using the popular names of the disease as a starting point.

## 1. Introduction

Leishmaniases are parasitic diseases common to humans and animals. They are caused by flagellated protozoa. These parasitoses pose serious public health problems in several countries around the world because of their great clinical and epidemiological diversity, the complexity of their parasite cycle, and the multiplicity of its reservoirs. In Morocco, these diseases occur in two forms: cutaneous leishmaniasis (CL) and visceral leishmaniasis (VL). They are considered a health priority, and their elimination should be achieved by 2030 [1].

From an epidemiological point of view, the World Health Organization (WHO) has classified Morocco among the 14 countries with a high burden of CL with an incidence rate of 5.62% and an at-risk population of 14%, while the incidence rate for VL was 0.91% with an at-risk population of 10% and a case-fatality rate of 2% [2]. Indeed, the visceral form, in Morocco, is considered as a zoonosis major, it is due to *Leishmania infantum* whose main vectors are *Phlebotomus perniciosus*, *Phlebotomus longicuspis*, and *Phlebotomus ariasi* and whose parasite reservoir is the dog [3, 4]. For cutaneous forms, in particular, CL to *Leishmania major*, it has been shown that the vector responsible for transmission was the *Phlebotomus papatasi*, and human infestation is from a single reservoir: Shawi meridians [4–6]. Concerning the CL in *Leishmania tropica*, it is a parasitosis whose vector is *Phlebotomus sergenti* and the reservoir is human. The infestation begins in midsummer and reaches its peak in early autumn [4, 6].

From the clinical point of view and concerning VL, the onset in children is silent and lasts 1 to 2 months, it manifests itself clinically as a general syndrome of fever, pallor, thinning of the lower limbs and chest contrasted with an increase in the volume of the abdomen, and a splenic-hepato-ganglionic syndrome (SHG), and other clinical signs may set in such as diarrhoea and thrombocytopenic purpura. Evolution is fatal in the absence of treatment. In contrast, the onset of visceral leishmaniasis in adults is abrupt, which manifests itself by febrile attacks, and an SHG syndrome is less clear-cut; the evolution is also severe but very slow [4].

Concerning the CL due to *Leishmania tropica*, also known as anthroponotic leishmaniasis, it is a disease found in arid and semiarid areas of Morocco. Its incubation period is from 20 to 7 months and it can reach 2 years, it starts with a lesion in the form of a red spot that quickly becomes a papule, painless, and itchy, this lesion is generally unique and located at the inoculation level, and the affected parts are those discovered in the body. During the period of the condition, the skin ulcerates in the centre of the papule, a yellowish liquid drains out and becomes a thickening crust, while the lesion enlarges to several centimetres in diameter. The evolution is very slow, it is done towards indelible healing [4].

For CL due to *Leishmania major*, also known as zoonotic cutaneous leishmaniasis, it is distinguished from the previous form by its more rapid evolution and by large lesions with a short incubation period of 10 to 45 days. The onset of clinical manifestations is characterized by an unglazed papule. The status period begins with ulceration of the lesions and rapid rounding to a diameter of 2 to 8 centimetres. The ulcer rests on an indurated base but does not adhere to the deep plane and is painless. The evolution results in disfiguring scars of 6 cm and within 6 months [4]. It should be noted that CL also exists in sporadic form due to *Leishmania infantum*. Indeed, it is a single ulcerous lesion on the face that can evolve for at least two years [4].

At present, the epidemiological situation of these parasitoses is worrying. According to the Moroccan Ministry of Health, the latest epidemiological surveillance data show that the number of new cases of leishmaniases was increasing every year with a predominance of the cutaneous form [1]. Also, 54% and 43% of the new leishmaniases cases registered, respectively, were CL due to *Leishmania major* and CL due to *Leishmania tropica*, and 3% of the new cases were CL due to *Leishmania infantum* [1].

To cope with this situation, the Ministry of Health has set up, since 1997, a National Leishmaniases Control Programme (NLCP) which is managed, at the central level, by the department of parasitic diseases control. However, despite the great efforts made, leishmaniases continue to spread in the country, and the proportion of cases detected at the level of health structures does not exceed 35% of the estimated cases [1].

Health professionals are in the best position to improve this figure, but this requires professional skills and knowledge about leishmaniases and the means to control it. This could increase the rate of case reporting and therefore speed up the process of elimination of these parasitoses. Indeed, the knowledge of health professionals plays an important role in the detection and recognition of the disease, in the awareness and education of the population to avoid risks related to the vector, reservoirs and to consult in case of clinical manifestations. However, no studies have been made of this professional population to know their state of knowledge and to explore their experiences in the field of screening and management of patients with leishmaniasis. Therefore, it is important to know the factors involved in the improvement of screening and management of leishmaniasis cases including the knowledge and experience of the caregivers.

This research aims to assess the knowledge of health professionals on leishmaniasis to determine the strengths and weaknesses in the control activities of these parasitoses at the local level and to identify the causes that are likely to lead to underreporting of cases. Also, this study is presented to unveil the knowledge of health professionals about leishmaniasis and explore their role in the peripheral management of the program and their experiences with leishmaniasis patients. The specific elements sought were: knowledge of leishmaniasis, vector, reservoirs, mode of transmission, experiences related to the local management of the national leishmaniasis control program, and management of leishmaniasis patients. The results will be of great importance for the decision-makers and managers of the NLCP insofar as they can understand the causes of underreporting of cases and subsequently implement corrective measures to accompany the development of professional skills in the fight against leishmaniases and accelerate their elimination process by 2030.

## 2. Materials and Methods

This is a cross-sectional exploratory study carried out between April and May 2019 among all medical and paramedical health professionals working in public health institutions in the province of El Hajeb which is located in central Morocco in the Fez-Meknes region where several leishmaniases outbreaks have recently been declared and other former ones have been reactivated [7, 8]. Also, a very recent study was carried out describing the epidemiological situation of leishmaniases in the ELHajeb province revealed that 75% of the newly reported cases of leishmaniases were indigenous people of rural origin, the coexistence of both cutaneous and visceral forms, the early age of human infestation which is 13 months for VL and 24 months for CL [9].

The study population was chosen to include all public health personnel (doctors, nurses, midwives) and health technicians (hygiene technicians and laboratory technicians) who are directly involved in health care activities. The province has 23 basic public health structures, including 12 communal health centres, 6 rural dispensaries and 5 urban health centres, and two hospital structures: a provincial hospital and a haemodialysis centre [10]. All of these structures were included as well as an ambulatory health centre reserved for the care and follow-up of leishmaniasis patients. These centres served a population of 247016 inhabitants, the majority of whom work in agriculture, animal husbandry, and food processing [11, 12].

Information was collected using a self-administered questionnaire containing direct, semidirect, and open-ended questions. This quantitative tool was designed to collect information on four themes: (a) the socioprofessional characteristics of the participants; (b) knowledge of the disease and the clinical manifestations of each form; (c) the vector, reservoirs, and mode of transmission; and (d) the experience of professionals with patients with CL, as the form responded to in the country, within the framework of the NLCP.

Before using this questionnaire, it was tested and validated in advance with fifteen health professionals working in the health structures of the city of Meknes and having socioprofessional characteristics similar to those of the population under study. The selection of participants was based on convenience sampling, and only those health professionals who agreed to answer the questionnaire were included. The interviews were conducted with the assistance of two teams of nursing and health technology students who are familiar with the city because of their frequent visits to its various health facilities during their professional internship periods and who are also well acquainted with field research and the conduct of surveys scientifically and ethically. Thus, the data obtained were entered into an Excel spreadsheet, checked, and transferred to Epi Info 7 for processing and statistical analysis. The results were presented in the form of rates, percentages, tables, and graphs.

However, all ethical considerations were respected in this study, as we initially obtained administrative authorization from the delegation of the Ministry of Health of the province of ELHajeb to conduct this research. Also, all health professionals participating in this study were informed about the objectives of the research and full respect for the confidentiality of their identities and responses. This left them completely free to accept or refuse to participate in the study as recommended by experts in scientific research ethics [13]. Respondents were thanked for their participation and were asked to respond as authentically as possible on all questions. As a result, the criteria for inclusion were acceptance to participate voluntarily in our study, and the existence in the care structures on the day the questionnaire was distributed. While the exclusion criteria were the refusal of professionals to participate in this study.

## 3. Results and Discussion

In Morocco, leishmaniases are a real public health problem, and they have been a notifiable disease since 1995. This study aims at documenting the knowledge of health workers on leishmaniosis and exploring their professional experiences in the management of patients suffering from these parasitoses. It is the first study, at the level of Morocco and the Maghreb, that has been carried out among people directly involved in the fight against leishmaniases.

The population under study includes all professional categories of caregivers under the delegation of the Ministry of Health of El Hajeb with rates similar to those of the national scale. Indeed, during the year 2018, the total number of medical and paramedical cadres of the Ministry of Health represents 25% and 64%, respectively [10]. At the provincial level, the total number of medical staff in El Hajeb is 248, distributed as follows: 22% of medical staff and 65% of paramedical staff [10]. Thus, 143 questionnaires were completed and collected from medical and paramedical health personnel during this study with a participation rate of 58%.

### 3.1. The Socioprofessional Characteristics of the Participants

The socioprofessional characteristics of the participants in terms of place of work, seniority in the health service, age, and gender are presented in Table 1. Analysis of these characteristics shows that the participation of the medical professional category is 29% (*n* = 41) and that of allied health professionals is 71% (*n* = 102). This is in perfect alignment with the proportions of each category at the provincial and national levels. These health professionals were probably representative of the Moroccan health care population [10].

In fact, 29% (*n* = 41) were doctors, 51% (*n* = 73) were nurses, 15% (*n* = 21) were midwives, and 6% (*n* = 8) were health technicians. Also, 63% of these health professionals worked in outpatient health facilities, 67% (*n* = 95) had 10 or more years of seniority in the health service, and females accounted for 53% (*n* = 76). Thus, this is a working population with median seniority of 10-15 years in the health services. This presupposes their accumulation of knowledge and professional experience in the operation of health programs and the care of the sick. This will increase our chances to have significant answers for the improvement of the management of patients suffering from leishmaniases and the management of NLCP.

### 3.2. Knowledge of the Leishmaniases Disease (CL and VL)

Table 2 summarizes the knowledge of caregivers about leishmaniases and the clinical manifestations of each form. One of the most important results is that 87% (*n* = 125) of the participants recognized the existence of leishmaniases in Morocco as a vector-borne disease and 41% among them consider that its current prevalence is increasing. This means, on the one hand, that the caregivers are aware of the epidemic situation of these diseases, and on the other hand, this corroborates with the epidemiological data on leishmaniases officially published by the Moroccan Ministry of Health and the World Health Organization [1, 2].

In Morocco, leishmaniases exist in two clinical forms: the cutaneous form and the visceral form. These two forms were recognized by our participants, and the analysis of the results showed that they identified the clinical manifestations of each form. Consequently, CL due to Leishmania tropica manifests most often as a single cutaneous lesion (62%) and dry (35%), and CL due to *Leishmania major* manifests as multiple cutaneous lesions (40%) and wet (6%). Whereas, visceral leishmaniasis (VL) is usually manifested by splenomegaly (73%), hepatomegaly (61%), and wasting (59%). These results are corroborated with those described by the WHO in the WHO expert report on the control of leishmaniases and the national guidelines for the control of leishmaniases in Morocco [4, 14].

### 3.3. The Knowledge of the Vector, the Reservoirs, and the Mode of Transmission

Skin forms are the most common throughout the country. They are caused by three different parasites and transmitted by different sand flies vectors. Also, CL due to *Leishmania tropica*, CL due to *Leishmania major*, and CL due to *Leishmania infantum* whose reservoirs are, respectively, man, rodent, and dog. And the visceral form is due to Leishmania infantum, which has as the confirmed reservoir of the parasite the dog [1]. The latter is the only known reservoir of VL in the Maghreb [15, 16]. And rodents are scientifically proven as natural reservoirs of zoonotic CL [17].

Our results regarding reservoirs are the most surprising (see Table 2). Health professionals do not differentiate between the reservoirs of each form of leishmaniases. Therefore, rodents, dogs, and humans are all designated as CL and VL reservoirs with percentage differences. Rodents were mentioned as reservoirs of CL (86%) and VL (74%). The dog was designated as a reservoir of VL (39%) and CL (22%). Humans were indicated as a reservoir for CL (12%) and VL (4%). These results show the flaccidity of caregivers' knowledge of the reservoirs, which can negatively influence the educational messages and awareness activities transmitted to the population and, consequently, the erroneous operationalization of the program axis devoted to IEC (Information, Education and Communication) of the population.

Concerning the vector, it is clear that sand flies are the only insects known to be vectors of leishmaniases [18, 19]. However, when the question was asked: “In your opinion, what is the vector agent of leishmaniases?” (see Table 2). Most caregivers mentioned more than two insects at the same time. They mentioned sand flies (56%), mosquitoes (54%), Anopheles (25%), flies (16%), and ticks (7%).

**Figure 1:**
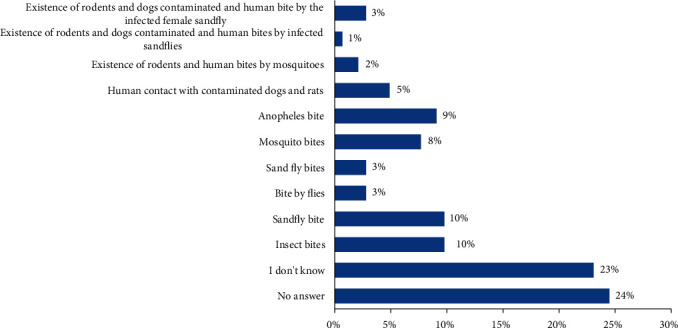
Modes of transmission of leishmaniases according to health professionals.

These discouraging results were also observed in the knowledge of the mode of transmission of the disease (Figure 1). This is although most health care workers have recognized leishmaniases as vector-borne disease; they are unable to specify how the disease is transmitted to humans. As a result, 24% responded with “I don't know,” and only 1% stated that the mode of transmission is through dogs and rodents infected and bitten by infected female sand flies in humans. The surprising fact was that some of them thought that the disease is transmitted to humans through contact with sick animals. Unfortunately, these misconceptions about the mode of transmission have also been observed among health professionals in Paraguay [20]. To remedy this situation, studies have suggested that the control of leishmaniases in Africa requires more understanding of the vector, reservoirs, and mode of transmission [21].

In short, these results reflect on the one hand the good knowledge of health professionals about the clinical signs of the cutaneous and visceral forms of leishmaniases, and on the other hand, the insufficient knowledge of health care workers about other aspects of the disease, the reservoir of each form, and their inability to know the true vector of leishmaniases. This will negatively influence the awareness and education activities for the population, either they did not carry them out because of the lack of knowledge, or they carried them out in an inappropriate way. This can be explained by the lack of continuous training in this subject or by the effects of malaria control activities as it is also a vector-borne disease where the control of the *anopheles* mosquito was the key element for the success of the national antimalaria program and the elimination of malaria in Morocco since 2012. Within this framework, it can be said that to succeed in the fight against leishmaniases, it is necessary to first organize continuous training sessions to inform and sensitize caregivers on the sand flies, its role in the transmission of the disease, and effective entomoprophylactic measures to avoid the risks of its bites, then broaden this awareness to include civil society actors to mobilize society to fight against leishmaniases and to educate the population, especially those exposed to the risks.

### 3.4. Knowledge and Experience of Health Professionals in the Peripheral Management of the National Leishmaniases Control Programme and the Management of Patients with CL

Due to their direct contact with the population in the health care services, the health care workers are in the best position to effectively control leishmaniasis. Their professional knowledge and skills in leishmaniases management, surveillance, and prevention are essential to eliminate these parasitoses. Describing the experiences of health workers in the management of leishmaniases and exploring the realities of operationalizing the objectives of the NLCP in the field will undoubtedly be very useful for managers to improve the performance of the program at the national level.

Health professionals' knowledge of the NLCP is presented in Table 3. Our results show that this program has been recognized by 76% (*n* = 108) of the interviewed health workers, and only 17% have received continuous training on leishmaniases control. This can be explained by the professional seniority of the respondents and the seniority of the program which exceeded 20 years. Also, 48% of the caregivers state that they have already treated/cared for a patient suffering from leishmaniasis, especially the cutaneous form (41%). Interestingly, only 25% of the caregivers reported that the scientific name of cutaneous leishmaniasis is different from the popular name of the disease in the population. Therefore, they said that this disease is known among the citizens by several names mainly the name “Hboub of Chniwla” (17%) and “Hboub of Namos” (9%). It should be noted that the concept of “Hboub” in the Moroccan dialect is used to designate a lesion that affects the dermis. These results confirm those proven by Bennis and his colleagues when they conducted a qualitative study on the psychological effects of CL in south-eastern Morocco in the localities of Tinghir and Errachidia, where they found that CL victims use the concepts of “Hboub of Chniwla” and “Hboub of Namos” to designate skin scars [22]. Therefore, it can be concluded that the popular names of the CL in Morocco do not differ between its central and southern regions. This said, using the popular names of CL as a starting point for educating citizens about CL will surely raise awareness among a large part of the Moroccan population, who will be able to easily know the lesions of CL (the form that causes it and accounts for 97% of leishmaniases cases in Morocco) and automatically seek care from health care providers, which will increase the rate of detection and case management.

The experiences of health professionals in the operationalization of the main objectives of the program are summarized in Table 4. 85% of the health workers opted for the objective of screening and therapeutic care, 33% for the objective of vector control, 42% for the objective of organizing information and continuing education days for health workers, 17% for the objective of organizing awareness and education days for the population, and 4% added intersectoral collaboration. The analysis of these results shows that the importance given to the achievement of each objective in the daily practice of the health personnel reflects the differences in the percentages recorded between the program objectives. Moreover, the majority of the NLCP objectives that are operationalized by health professionals focus on curative rather than preventive aspects. This is not in line with Boussa's remarks and the WHO recommendations, which stated that the preventive aspect is indispensable for a sustainable fight against leishmaniases [23, 24].

Moreover, the nonadherence to the antileishmaniasis treatment was confirmed by 47% of the health professionals (see Table 4). The causes of this attitude lie in the application of traditional treatments (36%) and the adverse effects of medical treatment (31%). Also, the health professionals argue that most citizens did not apply preventive measures (93%), which is related to the low socioeconomic level (78%) of the population and the lack of awareness of the risks related to insect bites (63%). These results are corroborated with those of Carmo and colleagues carried out in Brazil, where they revealed that there is a lack of information on VL among health professionals and a reluctance of patients to adhere to leishmaniasis prevention and control measures, especially about environmental management [25].

Thus, 33% of health professionals said that the NLCP program needs to be redesigned. Nevertheless, they made some recommendations to improve the control of leishmaniases and the performance of this program (see Table 5). Also, the professionals suggest the introduction of the use of rapid tests in VL diagnosis activities to increase the notification rate and the vaccination of canine reservoirs, as well as strengthening the skills of caregivers through the implementation of continuous training sessions and intersectoral collaboration at the peripheral level.

## 4. Conclusions

Currently, vector-borne diseases of parasitic origin such as leishmaniases are on the increase in many countries of the world including Morocco, the role of social actors directly involved in the fight against these parasitoses, and the recommendations discussed above are relevant in a wider context. In particular, one could focus on awareness-raising and use of rapid tests in the diagnosis of visceral leishmaniasis, adherence of patients to treatment, and retraining of health professionals by implementing continuing education sessions to correct some of the erroneous information, especially around the vector and reservoirs. Strengthening the skills of health care workers in this field, enabling them to properly educate the population to protect themselves against the risk of sand flies bites and to consult early on the appearance of leishmaniasis lesions. This will increase the case detection rate.

The study revealed that the scientific name of cutaneous leishmaniasis, which is the most common form of leishmaniasis in the country, is differently recognized by the citizens and therefore the popular name of the disease “Hboub of Chniwla”. This has been mentioned only by a few health professionals, which will explain the underreporting of cases at the level of health institutions, which does not exceed 35% at the national level. To remedy this situation, the implementation of public awareness programs should focus on informing people living in endemic areas about the mode of transmission, preventive measures against sand fly bites, and recognition of lesions using the popular names of the disease as a starting point. Such a strategy can help to increase the recognition of the disease by a large number of people and encourage those affected to seek treatment, which may lead to an increase in the detection rate and thus accelerate the process of eliminating leishmaniases.

This study concluded that among the strengths of the peripheral management of the NLCP is the good knowledge of health professionals of the curative aspect of the program and the clinical manifestations of each form of the disease. While among the most important weaknesses, we revealed the lack of knowledge about the true vector of leishmaniases, the reservoirs, and the mode of transmission, as well as the awareness and health education of the citizens, which is a secondary task. These results will, therefore, be of great importance for the decision-makers and managers insofar as they allow to target the key elements of the program that need to be strengthened to improve the fight against leishmaniases, especially the preventive aspect.

## Figures and Tables

**Table 1:** The socioprofessional characteristics of the participants.

Variable			*N* = 143	Percentage
Gender		M	67	47%
		F	76	53%
Age		20-30 years old	14	10%
		30-40 years old	68	48%
		40-50 years old	41	29%
		≥50 years old	20	14%
Working structure	Primary health care network	Doctors	26	18%
		Nurses	47	33%
		Midwife	15	10%
		Health technicians	2	1%
	Hospital network	Doctors	15	10%
		Nurses	26	18%
		Midwife	6	4%
		Health technicians	6	4%
Seniority in the health service		≤5 years old	6	4%
		5-10 years old	41	29%
		10-15 years old	54	38%
		15-20 years old	25	17%
		≥20 years old	17	12%

**Table 2:** Health professionals' knowledge of leishmaniases, vector, and reservoirs.

		Doctors (*n* = 41)	Nurses (*n* = 73)	Midwife (*n* = 21)	Health technicians (*n* = 8)	*N* = 143	Percentage
The clinical manifestations of cutaneous leishmaniasis	Single skin lesion	30	39	13	7	89	62%
	Multiple skin lesions	11	37	8	1	57	40%
	Dry skin lesions	13	32	5	0	50	35%
	Wet skin lesions	4	3	2	0	9	6%
	Other signs	3	3	0	0	6	4%
The clinical manifestations of visceral leishmaniasis	Splenomegaly	32	53	12	7	104	73%
	Hepatomegaly	25	42	14	6	87	61%
	Slimming	22	37	19	6	84	59%
	Fever	14	37	14	2	67	47%
	Hemorrhagic signs	4	5	0	0	9	6%
	I do not know	0	1	1	1	3	2%
The reservoir of cutaneous leishmaniasis	Rodents	41	61	14	7	123	86%
	Dog	9	12	9	2	32	22%
	Cat	5	5	7	0	17	12%
	Man	3	12	2	0	17	12%
	Livestock	0	4	2	1	7	5%
	Insect	3	1	1	0	5	4%
	I do not know	0	1	1	0	2	1%
The reservoir of visceral leishmaniasis	Rodents	33	53	14	7	107	75%
	Dog	16	31	6	3	56	39%
	Cat	5	6	5	0	16	11%
	Livestock	1	6	2	1	10	7%
	Man	2	2	1	0	5	4%
	I do not know	0	1	2	1	4	3%
The vector of leishmaniases	Sand flies	29	40	7	4	80	56%
	Mosquitoes	18	40	15	4	77	54%
	Anopheles	9	22	3	2	36	25%
	Flies	8	12	3	0	23	16%
	Ticks	0	9	1	0	10	7%

**Table 3:** Knowledge of the national program for the control of leishmaniases.

Question	Answer	*N* = 143	Percentage
Do you know the national leishmaniasis control program?	Yes	108	76%
	No	35	24%
Have you received continuous training in the control of leishmaniases?	Yes	24	17%
	No	119	83%
Based on your knowledge and experience, the current prevalence of leishmaniases is: (only one answer)	Low	22	36%
	Average	52	36%
	On the rise	59	41%
	No change	8	6%
	I do not know	2	1%
In your experience, is the leishmaniases control program: (only one answer)	Effective	60	42%
	Ineffective	14	10%
	Must be rethought	47	33%
	I do not know	22	15%
Have you ever treated or nursed a case of leishmaniases?	Yes	69	48%
	No	74	52%
If so, what is the type of this leishmaniases?	Cutaneous leishmaniasis	59	41%
	Visceral leishmaniasis	9	9%
	Mucocutaneous leishmaniasis	1	1%
In your knowledge and experience, how do citizens call cutaneous leishmaniasis?	Hboub of Chniwla	24	17
	Hboub of Namos	13	9%
	Hboub	5	3%
	Nar Lfarsiya	4	3%
	Bouchwika	3	2%
	Lichmaniyate	1	1%
	I do not know	93	65%

**Table 4:** Knowledge and experiences of health professionals in the operationalization of the program's objectives.

		Doctors (*n* = 41)	Nurses (*n* = 73)	Midwife (*n* = 21)	Health technicians (*n* = 8)	*N* = 143	Percentage
In your knowledge and experience, the main operational objectives of the national leishmaniases control program: (you may enter more than one answer)	Screening and therapeutic management of cases	38	16	17	5	121	85%
	Vector control	16	19	5	7	47	33%
	Fighting the reservoir	13	27	4	6	50	35%
	Education and public awareness	8	12	2	3	25	17%
	Continuing education for health professionals	21	30	5	4	60	42%
	Intersectoral collaboration	3	1	1	1	6	4%
In your experience, do patients adhere to antileishmaniasis treatment?	Yes	24	35	11	6	76	53%
	No	17	38	10	2	67	47%
In your opinion, what are the causes of nonadherence to leishmaniasis treatment?	Apply traditional treatments	12	31	7	2	52	36%
	Do not accept adverse effects of treatment	13	22	8	2	45	31%
	Do not understand the importance of treatment	5	8	7	1	21	15%
	Do not keep treatment appointments	4	7	3	0	14	10%
In your opinion, is the population applying preventive measures?	Yes	3	6	1	0	10	7%
	No	38	67	20	8	133	93%
In your experience, what are the causes that prevent citizens from applying preventive measures?	Ignorance of the risks associated with insects	26	47	11	6	90	63%
	Ignorance of preventive measures	27	31	9	5	72	50%
	Financial considerations	33	40	14	4	91	64%
	Low educational and sociocultural level	34	54	18	5	111	78%

**Table 5:** Suggestions from health professionals to improve the fight against leishmaniases in Morocco.

	*N* = 143	Percentage
Improved clinical and therapeutic case management		
(i) Continuous training of health professionals, especially in areas affected by leishmaniasis	41	29%
(ii) Introduction of the use of rapid tests for early diagnosis of VL and appropriate management	20	14%
(iii) Patient adherence to treatment	23	16%
Prevention		
(i) Promotion of health awareness and education of citizens through the media and new means of communication and social networks	57	40%
(ii) Vaccination of dogs and spraying of their habitat	28	20%
(iii) Reinforcement of vector and reservoir control	18	13%
(iv) Use of mosquito nets	9	6%
(v) Improving waste management	36	25%
(vi) Wearing long clothes	11	8%
Management		
(i) Collaboration between different sectors, cooperation with NGOs and associations, involvement and empowerment of the population	23	16%
(ii) Affection of dermatologists and hygiene technicians	5	3%
(iii) Implementation of a new control program and strategy	15	10%
(iv) Monitoring and evaluation	3	2%

## Data Availability

The data used in this study are included within the article.
